# Plasma Complement 3 and Complement 4 Are Promising Biomarkers for Distinguishing NMOSD From MOGAD and Are Associated With the Blood-Brain-Barrier Disruption in NMOSD

**DOI:** 10.3389/fimmu.2022.853891

**Published:** 2022-07-11

**Authors:** Liuyu Lin, Yuqing Wu, Hailun Hang, Jie Lu, Yuanliang Ding

**Affiliations:** Department of Neurology, The Affiliated Brain Hospital of Nanjing Medical University, Nanjing, China

**Keywords:** neuromyelitis optica spectrum disorder, myelin oligodendrocyte glycoprotein antibody-associated disease, complement 3, complement 4, blood brain barrier (BBB)

## Abstract

**Background and Objective:**

Neuromyelitis optica spectrum disorders (NMOSD) and myelin oligodendrocyte glycoprotein antibody (MOG-IgG) associated disease (MOGAD) are autoimmune inflammatory demyelinating diseases of the central nervous system (CNS). As the clinical features of NMOSD are similar to MOGAD, diagnostic confusion exists between the two diseases. To better discriminate NMOSD from MOGAD, we investigated whether the plasma levels of complement 3 (C3) and complement 4 (C4) are different in NMOSD and MOGAD during the acute attacks of the diseases. We sought to determine whether C3 or C4 has an influence on the features of NMOSD.

**Methods:**

In this observational study, data from 73 aquaporin-4 antibodies (AQP4-IgG) positive NMOSD patients and 22 MOG-IgG positive MOGAD patients were collected retrospectively. Demographics, clinical characteristics, plasma parameters, and cerebrospinal fluid (CSF) findings will be analyzed for comparability between the two groups. Immunoglobulin-G (IgG) and albumin were measured in both plasma and CSF. Plasma levels of C3 and C4 were measured and compared between the NMOSD, MOGAD, and 42 healthy controls (HC). The correlations between plasma C3, C4, and NMOSD clinical parameters were analyzed.

**Results:**

The ages of onset were later in the AQP4-IgG positive NMOSD group and females predominated, which differed from the MOGAD group, whose ages were younger and with a slight male preponderance. The AQP4-IgG positive NMOSD patients presented with the clinical symptoms of optic neuritis (ON) and transverse myelitis (TM), whereas encephalitis symptoms were more prevalent in MOGAD patients. CSF analysis shows that slight but not significantly higher white cell count (WCC) and protein were observed in the MOGAD group than in the AQP4-IgG positive NMOSD group. The plasma levels of IgG in MOGAD patients are significantly lower (*p* = 0.027) than in NMOSD patients. On the contrary, the plasma levels of albumin in MOGAD were higher than in NMOSD, which reached statistical significance (*p* = 0.039). Both the plasma C3 and C4 levels in the NMOSD group were significantly lower than in MOGAD and HC. The receiver operating characteristic (ROC) curve of the prediction model comprises C3 and C4 to distinguish NMOSD from MOGAD [area under the curve (AUC): 0.731, 0.645], which are considered to have discriminatory values. The results of Spearman’s analysis revealed that there was a significant positive correlation between the plasma C3 and the CSF WCC (r = 0.383, *p* = 0.040). There was an inverse correlation between plasma C4 and plasma IgG (r = -0.244, *p* = 0.038). Plasma C3 or C4 was significantly positively correlated with CSF albumin and Q-Alb, which is considered a measure of blood-brain barrier (BBB) disruption.

**Conclusion:**

During the acute phase of NMOSD and MOGAD, plasma C3 and C4 may become potential biomarkers for distinguishing the two diseases and reflecting the NMOSD BBB damage.

## Introduction

Neuromyelitis optica spectrum disorders (NMOSD) is a chronic, severe autoimmune demyelinating disease of the central nervous system (CNS) with the optic nerves and the spinal cord as primary target sites (1). It is widely accepted that the antibody target for the water channel aquaporin-4 (AQP4) is a pathogenic marker of NMOSD (2). Most NMOSD patients are seropositive for AQP4-IgG, but a proportion of NMOSD patients remain negative despite the use of cell-based assays (CBA) (3). In AQP4-IgG seronegative NMOSD, approximately 15% to 40% of myelin oligodendrocyte glycoprotein (MOG) antibody (MOG-IgG) are present (4, 5). Using the CBA, MOG-IgG was also detected in acute disseminated encephalomyelitis (ADEM), encephalitis, optic neuritis (ON), and myelitis (6). MOG-IgG-associated disease (MOGAD) has distinct biomarker, clinical, and radiologic characteristics from NMOSD and is considered an independent disease entity.

Experimental studies have indicated that AQP4-IgG primarily attacks the water channels of the astrocytes through antibody-dependent cellular cytotoxicity (ADCC) (7) or complement-dependent cytotoxicity (CDC) (8). The pathogenic mechanisms mediated by the complement system in MOGAD may be distinct from those in NMOSD (9). Since the activation of complement 3 (C3) and complement 4 (C4) is the core of complement activation, C3 and C4 are indispensable parts of the complement system (10). Previous studies showed plasma C3 (11) or C4 (12) was significantly lower in NMOSD compared to multiple sclerosis. Less focus has been given to the different levels of C3 and C4 in NMOSD and MOGAD. Therefore, the plasma C3, C4 levels be measured in AQP4-IgG positive NMOSD, MOGAD, and healthy controls (HC) to determine if there are any differences in AQP4-IgG or MOG-IgG associated effector mechanisms. Our study is also required to determine if there is a relationship between C3, C4 and the clinical features of NMOSD.

## Methods

### Samples

Patients who were recruited from the affiliated brain hospital of Nanjing Medical University between 2012 and 2021 were included in this single-center, retrospective observational study. Inclusion criteria are as follows: (1) be 18 years of age or older, and (2) be seropositive for AQP4-IgG by commercial CBA, meeting the IPND 2015 criteria (1) for NMOSD; (3) be seropositive for MOG-IgG by commercial CBA, meeting the Jarius et al. criteria (6) for MOGAD; (4) all plasma and CSF samples from NMOSD and MOGAD patients were in the acute phase of the diseases. Exclusion criteria included being seronegative for AQP4-IgG and MOG-IgG, having a disease that affects the complement system, or having missing clinical data. Overall, 73 NMOSD patients and 22 MOGAD patients met the inclusion and exclusion criteria and were included in this study. 42 healthy controls (HC) were recruited from the surrounding community. HC subjects suffering from neurological diseases or systemic autoimmune diseases were excluded. All patients will be included in this study after signing the informed consent. The study has been approved by the ethics committee of the affiliated Brain hospital of Nanjing Medical University.

### Data Collection

The demographic information, including gender and age at onset, was recorded. The clinical and paraclinical data were obtained from the electronic medical records and were compared between NMOSD and MOGAD. The clinical data included annual relapse rate (ARR), Expanded Disability Status Scale (EDSS) at sampling, clinical symptoms, and pharmacological treatments. The paraclinical data recorded included IgG and albumin, both in plasma and cerebrospinal fluid (CSF). CSF white cell count (WCC), CSF protein, oligoclonal bands, IgG index, CSF/plasma albumin ratio (Q-Alb), and plasma levels of C3, C4 were collected and analyzed. To assess the integrity of the blood-brain barrier (BBB), the CSF/plasma albumin ratio (Q-Alb) was considered a marker, and the detection of the IgG index was used as an indicator of intrathecal IgG synthesis. Plasma and CSF samples were obtained from NMOSD and MOGAD patients at the time of disease attack before hormone shock therapy. AQP4-IgG and MOG-IgG are also important indicators to distinguish NMOSD from MOGAD. The serum AQP4-IgG and MOG-IgG were all detected by a standardized immunohistochemical cell-based assay (CBA) according to the manufacturer’s protocol (Euroimmun, Germany) and using HEK293 cells transfected with human AQP4-M23 as a target to determine the titers. Plasma C3 and C4 were measured by immunofluorescence. Plasma levels of C3 (reference range, 0.90-1.80g/L) and C4 (reference range, 0.10-0.40g/L) were compared between AQP4-IgG-positive NMOSD, MOGAD and HC.

### Statistical Analysis

All statistical analysis was conducted in SPSS (version 24) software (SPSS, Inc., Chicago, IL) and GraphPad Prism 8.0 (GraphPad Software, Inc., San Diego, CA, USA). Differences in quantitative variables were compared using the student *t* statistics or Mann-Whitney U tests, while the chi-square test or Fisher exact probability methods were used for comparison of categorical variables. Comparisons between three groups (NMOSD group, MOGAD group, and HC group) were performed using a nonparametric Kruskal-Wallis-test. The corrected p-values using the Bonferroni correction to avoid type I errors. Receiver operating characteristics (ROC) curve analysis was used to calculate the area under the ROC curve (AUC) and evaluate the diagnostic value of plasma C3, C4 for differentiating NMOSD from MOGAD. To analyze correlations between plasma C3, C4 and EDSS, CSF findings, albumin and IgG both in plasma and CSF, IgG index, and Q-Alb, the Spearman correlation was used for correlation analysis.

## Results

### Demographic, Clinical Features, and Treatment in NMOSD and MOGAD Patients

A total of 73 AQP4-IgG positive NMOSD patients, 22 MOG-IgG positive MOGAD patients, and 42 HC were enrolled in this study. **Table 1** shows the demographics, ARR, disease duration, EDSS, clinical symptoms, and immunotherapy treatment in the NMOSD and MOGAD groups. Compared to MOGAD, there was a female gender dominance of the NMOSD participants (94.52% *vs.* 27.27%, *p*<0.001). The age at onset in the NMOSD group is slightly older than in the MOGAD group, but has not reached statistical significance. There was no significant difference between NMOSD and MOGAD regarding ARR, disease duration, or EDSS. Significance differences were found between the NMOSD and MOGAD groups regarding the clinical symptoms. In summary, more optic neuritis (ON, 57.53% *vs.* 31.82%, *p* = 0.034) and transverse myelitis (TM, 75.34% *vs*. 31.82%, *p*<0.001) present in NMOSD, whereas acute disseminated encephalomyelitis (ADEM, 9.59% *vs.* 50%, *p*<0.001) is common in MOGAD.

**Table 1:** The detailed findings of Demographic, clinical features, and treatment in NMOSD and MOGAD patients.

	HC	NMOSD (AQP4-IgG positive)	MOGAD (MOG-IgG positive)	*p* ^a^	*p* ^b^	*p* ^c^
Patients number	42	73	22			
Female, n (%)	28, (66.67)	69, (94.52)	6, (27.27)	0.003*	0.002*	<0.001*
Age at onset (mean ± SD)	42.07 ± 9.42	41.86 ± 14.27	37.91 ± 17.26	0.925	0.302	0.336
ARR, median, (IQR)	–	0.8, (0.16-1.32)	0.83, (0.43-1.21)	–	–	0.876
Disease duration, years, median, (IQR)	–	4, (1-9)	2, (1-2)	–	–	0.175
EDSS, median, (IQR)	–	3.0, (2.0-4.0)	3.0, (2.0-4.0)	–	–	0.775
Clinical symptom, n (%)						
ON	–	42, (57.53)	7, (31.82)	–	–	**0.034**
TM	–	55, (75.34)	7, (31.82)	–	–	**<0.001**
Encephalopathy	–	7, (9.59)	11, (50)	–	–	**<0.001**
Acute treatment, n (%)						
IVMP	–	71, (97.26)	21, (95.45)	–	–	0.551
IVIg	–	24, (32.88)	6, (27.27)	–	–	0.795
PLEX	–	1, (1.37)	0, (0)	–	–	1.000
Maintenance treatment, n (%)						
Mycophenolate mofetil		26, (35.62)	9, (40.91)	–	–	0.801
Azathioprine		11, (15.07)	1, (4.55)	–	–	0.284
Cyclosporine		1, (1.37)	0, (0)	–	–	1.000
rituximab		1, (1.37)	0, (0)	–	–	1.000

HC, healthy control; AQP4-IgG, aquaporin-4 immunoglobulin-G; NMOSD, neuromyelitis optica spectrum disorders; MOG-IgG, myelin oligodendrocyte glycoprotein immunoglobulin-G; MOGAD, MOG-IgG-associated disease; ARR, annual relapse rate; EDSS, Expanded Disability Status Scale; SD, standard deviation; IQR, interquartile range; ON, optic neuritis; TM, transverse myelitis; IVMP, intravenous methylprednisolone; IVIg, intravenous immunoglobulins; PLEX, plasma exchange.

a AQP4-IgG positive NMOSD versus HC.

b MOGAD versus HC.

c AQP4-IgG positive NMOSD versus MOGAD.

bold: p<0.05; *: Bonferoni-adjusted p<0.05.

In our cohort, receipt of a high dose of corticoid medication was found in all NMOSD and MOGAD patients during the acute-phase. Most NMOSD (97.21%) and MOGAD (95.45%) patients were started at a dose of 500mg/d and gradually tapered, maintaining a dose of 10–15 mg/d in remission. In addition to hormone shock therapy, some patients use intravenous immunoglobulins (IVIg) or plasma exchange (PLEX) during the acute phase to improve treatment efficacy. The use of immunosuppressants is associated with slow disease progression and reduced relapse in patients who have had CNS demyelinating diseases. In our cohort, mycophenolate mofetil was the most frequently used immunosuppressant in remission, followed by azathioprine. Eleven NMOSD patients and one MOGAD patient had been treated with Azathioprine. The other two NMOSD patients in the study had received cyclosporine and rituximab, respectively.

### Comparison of Plasma and CSF Detailed Findings Among Patients With NMOSD and MOGAD Patients

**Figure 1:**
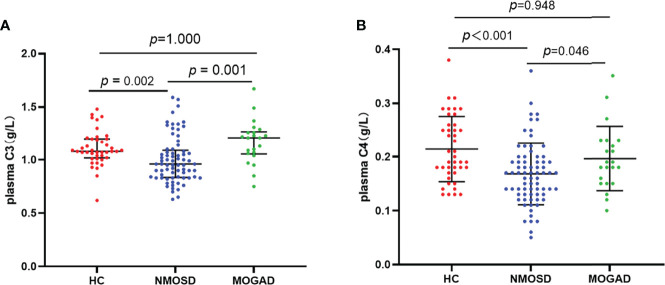
Plasma levels of C3 **(A)** and C4 **(B)** in heathy control, AQP4-IgG positive NMOSD, and MOGAD. Results are presented as scatter plots with the median and interquartile range (IQR). The statistically significant differences were analyzed using the Kruskal-Wallis-test and the corrected p-values using the Bonferroni. C3, complement 3; C4, complement 4; HC, healthy control; NMOSD, neuromyelitis optica spectrum disorders; MOGAD, myelin oligodendrocyte glycoprotein antibody associated disease.

**Figure 2:**
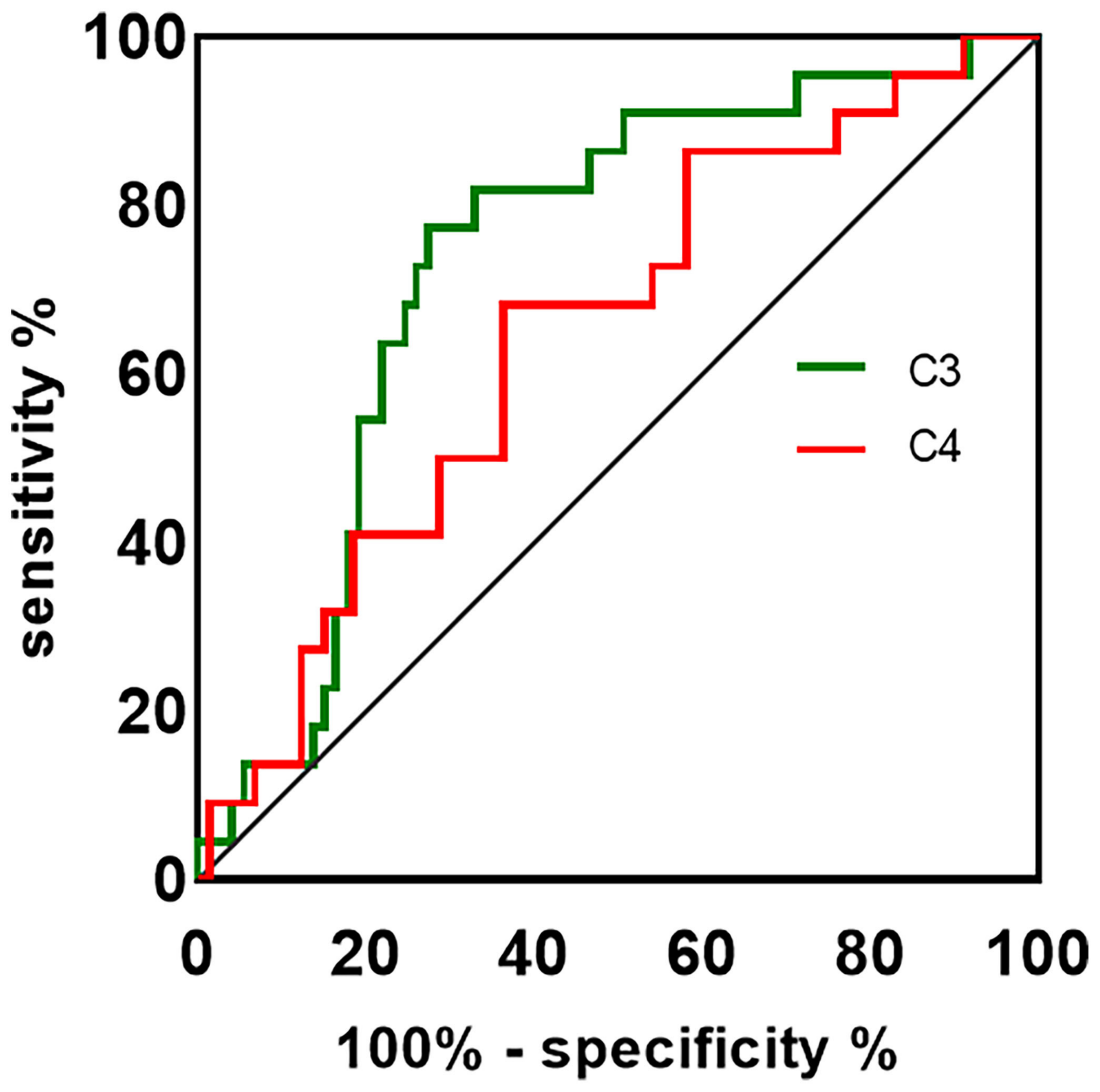
The ROC curve is used to assess the discriminating ability of plasma C3, C4 in NMOSD and MOGAD. The AUC values showed the predictive power of the C3 (0.731) and C4 (0.645). ROC, receiver-operating characteristic; AUC, area under curve; C3, complement 3; C4, complement 4.

Detection of antibodies by the CBA test allowed quantitative measurements of AQP4-IgG and MOG-IgG serum titers. The median AQP4-IgG titer is 1:100 (IQR: 1:32-1:320) and the MOG-IgG titer is 1:10 (IQR: 1:10-1:100). **Table 2** summarizes the plasma and CSF detailed findings of the study subjects. In this study, the CSF samples of 29 NMOSD and 17 MOGAD patients were collected and analyzed before any treatment. In terms of findings of WCC, protein, and oligoclonal bands positivity in CSF, no significant difference could be detected between patients with NMOSD and MOGAD groups. Significant differences were also not observed in the comparison of the two groups regarding Q-Alb and the IgG index. Regardless of the slight differences in levels of IgG and albumin in CSF, there were significant differences in plasma (*p* = 0.027 and *p* = 0.039, respectively). The NMOSD group plasma level of C3, C4 was lower in the HC and MODAD groups, where the difference reached statistical significance. There were no significant differences in plasma levels of C3, C4 in MOGAD and HC. The differences in levels of plasma C3, C4 in HC, NMOSD, and MOGAD groups were presented as scatter dot plots, which had medians and interquartile ranges (IQR) (**Figure 1A, B**). Receiver-operating characteristic (ROC) analysis indicated that plasma C3, C4 dramatically distinguished NMOSD from MOGAD (**Figure 2**). The models to differentiate between NMOSD and MOGAD were evaluated by area under curve (AUC). Plasma C3 and C4 had AUCs of 0.731 and 0.645, respectively, which was considered moderately predictive.

**Table 2:** Plasma and CSF detailed findings among patients with NMOSD and MOGAD patients.

	HC	NMOSD (AQP4-IgG positive)	MOGAD (MOG-IgG positive)	*p* ^a^	*p^b^*	*p^c^*
AQP4-IgG titer, median, (IQR)	–	1:100(1:32-1:320)	–	–	–	–
MOG-IgG titer, median, (IQR)	–	–	1:10(1:10-1:100)	–	–	–
CSF findings		n=29	n=17
WCC, cell/μL, median, (IQR)	–	8, (4-20),	15, (6-108),	–	–	0.056
Protein, mg/dL, median, (range)	–	59, (42-77)	64, (44-74)	–	–	0.724
Oligoclonal bands positivity, n (%)	–	0, (0)	1, (4.55)	–	–	0.232
CSF IgG, (mg/L) median, (IQR)		49.1, (30-74)	37, (30.7-84.5)			0.937
CSF albumin, median, (IQR)	–	257, (174-385)	310, (264-405)			0.125
Plasma IgG, (mg/L) median, (IQR)	–	11.9, (8.95-14.2)	9.26, (7.81-11.75)			**0.027**
Plasma albumin, median, (IQR)	–	39.4, (37-42.2)	41.4, (39.2-43.5)			**0.039**
IgG index	–	0.62, (0.44-0.82)	0.62, (0.57-0.68)			0.794
Q-Alb, median, (IQR),	–	7.18, (4.38-9.62)	7.16, (5.99-10.17)			0.393
C3(g/L), median, (IQR)	1.08, (1.03-1.20)	0.96, (0.84-1.08)	1.21, (1.06-1.26)	0.002*	1.000	0.001*
C4(g/L), median, (IQR)	0.20, (0.17-0.26)	0.17, (0.13-0.19)	0.19, (0.15-0.23)	<0.001*	0.948	0.046*

HC, healthy control; AQP4-IgG, aquaporin-4 immunoglobulin-G; NMOSD, neuromyelitis optica spectrum disorders; MOG-IgG, myelin oligodendrocyte glycoprotein immunoglobulin-G; MOGAD, MOG-IgG-associated disease; CSF, cerebrospinal fluid; WCC, white cell count; IgG, immunoglobulin-G; IQR, interquartile range; Q-Alb, CSF/plasma albumin ratio; C3, complement 3; C4, complement 4.

a AQP4-IgG positive NMOSD versus HC.

b MOGAD versus HC.

c AQP4-IgG positive NMOSD versus MOGAD.

bold: p<0.05; *: Bonferroni-adjusted p<0.05.

### Correlations Between Plasma C3, C4 and Clinical Features of NMOSD Patients

**Figure 3:**
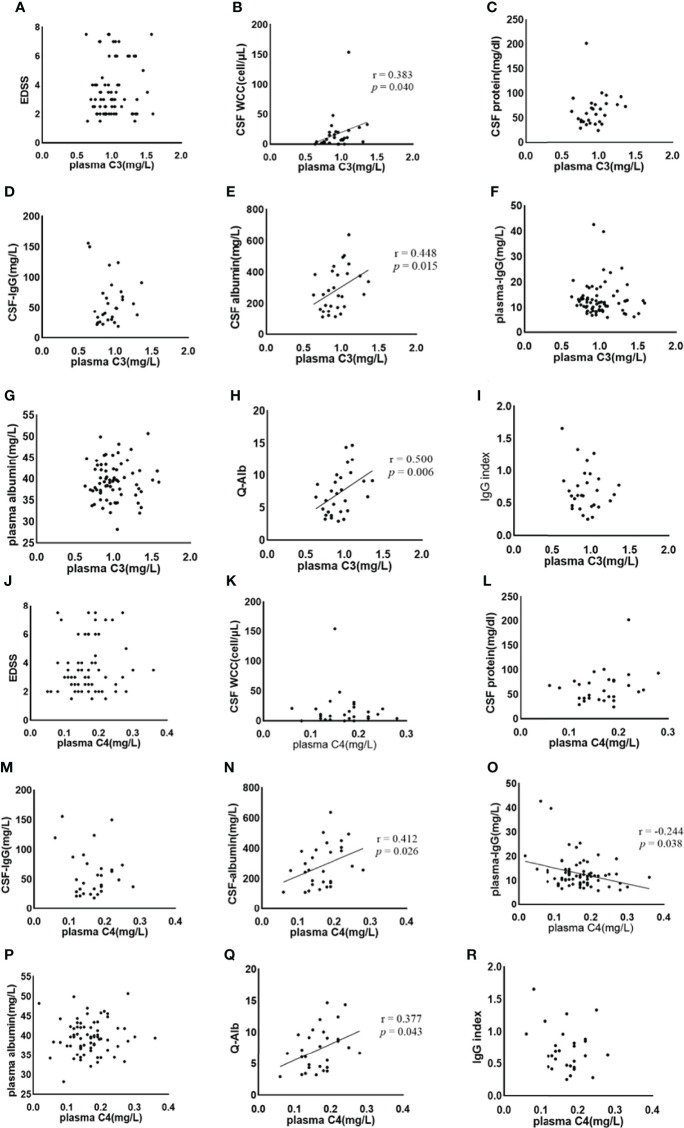
The association between plasma C3, C4 and NMOSD features was analysed by Spearman’s correlation. C3, complement 3; C4, complement 4; EDSS, Expanded Disability Status Scale; CSF, cerebrospinal fluid; WCC, white cell count; IgG: immunoglobulin-G; Q-Alb, CSF/plasma albumin ratio. The **(A–I)** shows the Spearman results of serum complement C3 and EDSS **(A)**, CSF WCC **(B)**, CSF protein **(C)**, CSF-IgG **(D)**, CSF albumin **(E)**, plasma IgG **(F)**, plasma albumin **(G)**, Q-Alb **(H)**, and IgG index **(I)**, respectively. There is a significant association between plasma CSF WCC, CSF albumin, and Q-Alb. The **(J–R)** shows the Spearman results of serum complement C4 and EDSS **(J)**, CSF WCC **(K)**, CSF protein **(L)**, CSF-IgG **(M)**, CSF albumin **(N)**, plasma IgG **(O)**, plasma albumin **(P)**, Q-Alb **(Q)**, and IgG index **(R)**, respectively. Positive associations exist between plasma C4 and CSF albumin, and Q-Alb respectively. A negative association exists between plasma C4 and plasma IgG.

Spearman’s correlation was used to analyze the correlation between plasma C3, C4, and EDSS, the plasma level of IgG, and albumin. The correlation between the plasma C3, C4, and CSF findings (WCC, protein, IgG, and albumin), Q-Alb, and IgG index were also examined by Spearman correlation analysis. Negative correlations were much less common than positive correlations. The results of the Spearman correlation analysis are shown in **Table 3**. Plasma C3 and C4 do not correlate with EDSS (**Figures 3A, J**, respectively), CSF-protein (**Figures 3C, L**, respectively), CSF-IgG (**Figures 3D, M**, respectively), plasma albumin (Figures 3G, P, respectively), and IgG index (**Figures 3I, R**, respectively). Similar unmeaningful results were attained when the correlation between Plasma C4 and CSF-WCC was examined (**Figure 3K**). A significant association between plasma C3 and CSF-WCC was found (r = 0.383, *p* = 0.040, **Figure 3B**). However, no significant association was found between plasma C3 and plasma-IgG (**Figure 3F**). The results of the analysis show a correlation between plasma C4 and plasma IgG (r = -0.244, *p* = 0.038, **Figure 3O**). Plasma C3 significantly correlates with CSF albumin (r = 0.448, *p* = 0.015, **Figure 3E**), and Q-Alb (r = 0.500, *p* = 0.006, **Figure 3H**). Similarly, it was found that good correlations exist between plasma C4 and CSF albumin (r = 0.412, *p* = 0.026, **Figure 3N**), and Q-Alb (r = 0.377, *p* = 0.043, **Figure 3Q**).

**Table 3:** Correlations between plasma C3, C4, and EDSS, immunological findings of NMOSD patients.

Clinical findings	C3		C4	
	Correlation coefficient	*p*-value	Correlation coefficient	*p*-value
EDSS	0.093	0.433	0.158	0.183
CSF-WCC	0.383	**0.040**	0.073	0.704
CSF protein	0.311	0.100	0.249	0.192
CSF-IgG	0.147	0.445	0.049	0.803
CSF albumin	0.448	**0.015**	0.412	**0.026**
plasma IgG	-0.096	0.421	-0.244	**0.038**
plasma albumin	0.009	0.943	0.033	0.783
Q-Alb	0.500	**0.006**	0.377	**0.043**
IgG index	-0.063	0.742	-0.121	0.532

C3, complement 3; C4, complement 4; EDSS, Expanded Disability Status Scale; CSF, cerebrospinal fluid; WCC, white cell count; IgG, immunoglobulin-G; Q-Alb, CSF/plasma albumin ratio; bold: p<0.05.

## Discussion

Our study also provides evidence that NMOSD and MOGAD are two distinct diseases, as reflected by demographic, clinical, and molecular data. In our cohort, female patients account for 94.5% of all NMOSD patients, while female patients only account for 27.3% of all MOGAD. Discrepancies in the male to female incidence ratio in the MOGAD group from the previous study are likely due to the low sample size (13). The age onset was not compatible among the NMOSD and MOGAD groups. We saw a preference for AQP4-IgG positive NMOSD in the ON and TM, while ADEM was more likely observed in MOGAD, which is in line with previous research (14).

Compared to the plasma median of the MOG-IgG titer, the AQP4-IgG titer is higher, these discrepancies could be due to different sensitivity of assay methods. In a multicenter study, although the CBA detect the MOG-IgG showed excellent agreement with other assays for both highly positive and negative samples, the low positive remained (15). NMOSD patients show significantly higher plasma immunoglobulin (IgG) than MOGAD patients (*p* = 0.027), which may reflect the different molecular mechanisms that exist in the two diseases. *In vitro in vivo* findings have shown that when reaching serum AQP4-IgG titers in experimental animals comparable with NMOSD patients, they are sufficient to trigger immune cascade reactions (16), while only the affinity-purified MOG-IgG extracted from MOGAD has the potential to cause pathogenicity (17). Therefore, we speculated that the CNS is more susceptible to inflammatory attack by AQP4-IgG than MOG-IgG. In our cohort study, plasma levels of albumin in NMOSD were significantly lower than in MOGAD. Yao et al. (18) reported that the low level of plasma albumin is associated with more disease severity in NMOSD. A possible explanation for our result as follows: Albumin has the potential to regulate immunology, anti-inflammatory function, and its decrease has also been described as associated with increased systemic inflammatory load (19). This implies a more vigorous inflammatory load produced by NMOSD.

Compelling evidence also shows that different underlying autoimmune-driven mechanisms exist in the NMOSD and MOGAD, in which the complement system plays an important role (20, 21). There is substantial evidence that complement analytes have been proved to distinguish MS from NMOSD (22). However, there are comparatively few studies on whether the levels of the complement components are different in NMOSD and MOGAD. C3, C4 are the central component of complement, and C3 is the convergent point of all the complement activation pathways (classical pathway, lectin pathway, or alternative pathway). C3,C4 are key complement factor in the complement activation pathway and reacts with C5, C6, C7, C8, and C9, participate in the formation of membrane attack complex (MAC, C5b-9), which acts as a permeability pore to cause astrocyte injury, followed by the BBB disruption, myelin loss, gliosis, and neuronal death in NMOSD (23). Our results indicated that the plasma levels of C3, C4 in AQP4-IgG positive NMOSD patients were lower than MOGAD and reached statistical significance, these results tallied with previous studies (12, 24). No statistical difference was observed in the C3, C4 between the MOGAD and HC. From the ROC curve analysis, conclusions can be drawn regarding C3, C4, which can become discriminating factors for NMOSD and MOGAD. Perhaps our findings can be explained by the subtle consumption of C3, C4 in NMOSD rather than MOGAD. MOG-IgG targeted the oligodendrocytes (MOG expressed on the myelin sheath) to activate the complement system, resulting in myelin loss but relative axonal and astrocyte preservation (9). The specific mechanism of complement system mediated-injury in MOGAD needs to be studied and further elucidated.

Our results show that plasma C4 is negatively correlated with plasma-IgG, maybe the more IgG is produced, in particular AQP4-IgG, the more plasma C4 consumption. The negative correlation between C3 and plasma IgG may indicate that C3 was consumed in the synthesis of IgG (maybe AQP4-IgG). However, the relationship between C3-the convergent point of all the complement activation pathways and plasma IgG is not simply linear or nonlinear. When C3 is cleaved, it releases C3a, C3b and C3d and binds to CR1 and CR2, which are expressed in the B cells. CR1 binds C3b with high affinity to inhibit B cell receptors (BCR), which mediate B cell activation, proliferation, and antibody production. The inhibitory effects of CR2 and C3d binding on the initial steps of peripheral B cell activation play a significant role in the maintenance of peripheral B cells (25). This suggests that C3 may have a natural feedback mechanism to keep activated B cells from producing too many antibodies. Most NMOSD are seropositive for IgG1 autoantibodies against AQP4 (26), followed by the binding of AQP4 to activate CDC and ADCC mediated astrocyte injury (27). In NMOSD, there may be IgG subclass that is similar to MOGAD (MOG-IgG 1,2,3,4) (28). The IgG4 subclass in NMOSD may have limited ability to mobilize CDC and ADCC and blocks the ligand-receptor interaction of the target antigen (29). Therefore, the relationship between C3 and plasma IgG in NMOSD is complicated, and further experiments will be necessary to clarify the mechanisms involved.

Both C3 and C4 significantly correlate with CSF albumin and Q-Alb. The Q-Alb is considered a common indicator for the evaluation of the destruction of the BBB. Abundant evidence supports a pathogenesis mechanism for AQP4-IgG positive NMOSD in which AQP4-IgG binds to the astrocyte endfoot of AQP4 to activate CDC *via* the classical pathway by binding to C1q, which is followed by C3 convertase enzymes converting C3 into C3a and C3b (20). Once cleaved, C3a sends signals and binding to its receptor C3aR, which is expressed by the vascular endothelial cells in the brain (30), resulting in the altered vascular morphology and increased BBB permeability (31). Therefore, we propose that C3 indirectly affects the BBB through the C3aR binding to C3a, which is elevated in the plasma of NMOSD (32). Increased permeability of the BBB caused by complement activation can also explain the massive infiltration of leucocytes in CSF (33). There is also evidence from a recent study that C3 is elevated in NMOSD CSF compared to controls (22), implying blood-cerebrospinal fluid barrier dysfunction, which leads to C3 entering into CSF and then affecting the immune environment and leukocytes in CSF. Plasma C3 positively correlates with the CSF-WCC, proposing the view that C3 has an impact on the CSF-WCC through increased blood-cerebrospinal fluid and BBB permeability. The regulation mechanism of C4 in the BBB of NMOSD patients remains elusive. C4a, is released from complement component C4 upon activation of the complement system’s classical and lectin pathways. C4a-induced activation of the protease-activated receptors 1 and 4 (PAR1, PAR4) has an impact on the stability of endothelial cells, thereby increasing the BBB permeability (34). Although the elevated C4 in CSF of NMOSD (22), it may be that the levels of plasma C4 entering the CNS through the blood-cerebrospinal fluid barrier and the plasma C4 are not enough to cause the change in WCC.

It is important to note that there are differences in the effects of C3, and C4 in SLE. In SLE, low complement (C3, C4) is an important serological manifestation. According to a previous study, C3 has a negative relationship with IFN- and IL-18, which have the highest positive likelihood ratios for active SLE (35). Durcan et al. (36) found there is a strong relationship between low C3 and lupus nephriand that is associated with poor renal outcomes (glomerular filtration rate (GFR) <50 and chronic proteinuria). C4 did not seem to affect disease activity and lupus nephritis. The discrepancy between C3 and C4 is, we propose, a more important marker due to the C3’s central role in the complement cascade, and the fact that complement components or complement activator molecules are released and play different roles in autoimmune diseases.

The present study suffers from several limitations. The MOGAD group sample size was small compared to the NMOSD group, which might have yielded statistical bias. In addition, they may be prone to recall bias because of the retrospective nature of the study. Moreover, all the participants are ethnically Chinese, so our results may yield different results and may not apply to other countries. Although the plasma levels of C3, C4 are found to be lower in NMOSD than in MOGAD, the mechanics of these factors were explored less deeply during the study. Further studies about the complement system in the pathogenesis of mediated tissue injury in CNS inflammatory demyelinating disorders need to be performed and to elaborate on the specific mechanisms more deeply and accurately.

## Conclusion

Our study reveals that there seems to be more plasma C3, C4 consumption in the NMOSD, further implying that the plasma C3, C4 can be able to distinguish the NMOSD from MOGAD. Plasma C3 or C4 may become potential biomarkers reflecting BBB disruption in NMOSD.

## Data Availability Statement

The original contributions presented in the study are included in the article/supplementary material. Further inquiries can be directed to the corresponding author.

## Ethics Statement

The studies involving human participants were reviewed and approved by The Ethics Committee of the Affiliated Brain Hospital of Nanjing Medical University. Written informed consent to participate in this study was provided by the participants’ legal guardian/next of kin. Written informed consent was obtained from the individual(s), and minor(s)’ legal guardian/next of kin, for the publication of any potentially identifiable images or data included in this article.

## Author Contributions

LL participated in the conceptualization, collected and analyzed the data, and wrote the original manuscript. YW proofread the data and analyzed the data again. HH visualized the data. JL and YD reviewed and suggested the manuscript and provided financial support. All authors approved the final manuscript before submission.

## Funding

The research is financially sponsored by the National Natural Science Research Foundation of China (81500969).

## Conflict of Interest

The authors declare that the research was conducted in the absence of any commercial or financial relationships that could be construed as a potential conflict of interest.

## Publisher’s Note

All claims expressed in this article are solely those of the authors and do not necessarily represent those of their affiliated organizations, or those of the publisher, the editors and the reviewers. Any product that may be evaluated in this article, or claim that may be made by its manufacturer, is not guaranteed or endorsed by the publisher.
